# CDKL5 deficiency-related neurodevelopmental disorders: a multi-center cohort study in Italy

**DOI:** 10.1007/s00415-024-12421-1

**Published:** 2024-06-14

**Authors:** Giovanni Battista Dell’Isola, Antonella Fattorusso, Francesco Pisani, Mario Mastrangelo, Duccio Maria Cordelli, Piero Pavone, Pasquale Parisi, Alessandro Ferretti, Francesca Felicia Operto, Maurizio Elia, Marco Carotenuto, Dario Pruna, Sara Matricardi, Elisabetta Spezia, Alberto Spalice, Giovanna Scorrano, Salvatore Savasta, Paolo Prontera, Giuseppe Di Cara, Daniela Fruttini, Vincenzo Salpietro, Pasquale Striano, Alberto Verrotti

**Affiliations:** 1 Department of Pediatrics, University of Perugia, Perugia, Italy; 2 Child Neurology and Psychiatry Unit, Department of Human Neurosciences, Sapienza University of Rome, 00185 Rome, Italy; 3 Department of Women/Child Health and Urological Science, Sapienza University of Rome, Rome, Italy; 4 UOC Neuropsychiatry of the Pediatric Age, IRCCS Institute of Neurological Sciences of Bologna, Bologna, Italy; 5 Section of Pediatrics and Child Neuropsychiatry, Department of Clinical and Experimental Medicine, University of Catania, Catania, Italy; 6 Pediatrics Unit, Neuroscience, Mental Health and Sense Organs (NESMOS) Department, Faculty of Medicine and Psychology, Sapienza University of Rome, 00189 Rome, Italy; 7 Child and Adolescent Neuropsychiatry Unit, Department of Medicine, Surgery and Dentistry, University of Salerno, Salerno, Italy; 8 Department of Science of Health, School of Medicine, University Magna Graecia of Catanzaro, Catanzaro, Italy; 9 Unit of Neurology and Clinical Neurophysiopathology, Oasi Research Institute-IRCCS, Troina, Italy; 10 Clinic of Child and Adolescent Neuropsychiatry, Department of Mental Health, Physical and Preventive Medicine, Università degli studi della Campania ‘Luigi Vanvitelli’, Naples, Italy; 11 Child Neurology and Epileptology Unit, Paediatric Department, ARNAS Brotzu, Cagliari, Italy; 12 Department of Pediatrics, University of Chieti, Chieti, Italy; 13 Pediatric Neurology, Azienda Policlinico Modena, Modena, Italy; 14 Department of Pediatrics, “Sapienza” University of Rome, Rome, Italy; 15 Department of Biotechnological and Applied Clinical Sciences, University of L’Aquila, L’Aquila, Italy; 16 Pediatric Clinic and Rare Diseases, Microcythemic Pediatric Hospital “A. Cao”, University of Cagliari, Cagliari, Italy; 17 Medical Genetics Unit, Hospital Santa Maria Della Misericordia, Perugia, Italy; 18 Department of Medicine and Surgery, University of Perugia, Perugia, Italy; 19 Department of Neuromuscular Diseases, Queen Square Institute of Neurology, University College London, London, UK; 20 Giannina Gaslini Institute, Scientific Institute for Research and Health Care, Genoa, Italy; 21 Department of Neurosciences, Rehabilitation, Ophthalmology, Genetics, Maternal and Child Health, University of Genoa, Genoa, Italy

**Keywords:** CDKL5 deficiency disorder, Hypermotor-tonic-spasms sequence seizures, Antiseizure medication, EEG, Cortical blindness

## Abstract

CDKL5 deficiency disorder (CDD) is a complex clinical condition resulting from non-functional or absent CDKL5 protein, a serine–threonine kinase pivotal for neural maturation and synaptogenesis. The disorder manifests primarily as developmental epileptic encephalopathy, with associated neurological phenotypes, such as hypotonia, movement disorders, visual impairment, and gastrointestinal issues. Its prevalence is estimated at 1 in 40,000–60,000 live births, and it is more prevalent in females due to the lethality of germline mutations in males during fetal development. This Italian multi-center observational study focused on 34 patients with CDKL5-related epileptic encephalopathy, aiming to enhance the understanding of the clinical and molecular aspects of CDD. The study, conducted across 14 pediatric neurology tertiary care centers in Italy, covered various aspects, including phenotypic presentations, seizure types, EEG patterns, treatments, neuroimaging findings, severity of psychomotor delay, and variant-phenotype correlations. The results highlighted the heterogeneity of seizure patterns, with hypermotor-tonic-spasms sequence seizures (HTSS) noted in 17.6% of patients. The study revealed a lack of clear genotype–phenotype correlation within the cohort. The presence of HTSS or HTSS-like at onset resulted a negative prognostic factor for the presence of daily seizures at long-term follow-up in CDD patients. Despite extensive polypharmacotherapy, including medications such as valproic acid, clobazam, cannabidiol, and others, sustained seizure freedom proved elusive, affirming the inherent drug-resistant nature of CDD. The findings underscored the need for further research to explore response rates to different treatments and the potential role of non-pharmacological interventions in managing this challenging disorder.

## Introduction

CDKL5 deficiency disorder (CDD) is a complex group of clinical conditions resulting from the presence of non-functional or absent CDKL5 protein, a serine–threonine kinase encoded by the CDKL5 gene (MIM: 300203) and implicated in neural maturation and synaptogenesis processes [1]. Developmental epileptic encephalopathy 2 (DEE2, MIM: 300672) (DEE2, MIM: 300672) represents one of the main clinical presentations of CDD and is characterized by severe neurodevelopmental impairment and refractory seizures [2, 3]. Other neurological phenotypes and/or comorbidities associated with pathogenic variants in *CDLK5* include hypotonia, sleep disturbances (due to obstructive airway symptoms and central apnea), hyperkinetic movement disorders (*i.e.*, dyskinesias, chorea and/or choreathoethosis), motor (hand-predominant) stereotypies, cortical–visual impairment (CVI) with early loss of eye fixation, dysphagia, and other gastrointestinal problems [4, 5].

The prevalence of pathogenic variants in *CDKL5* is estimated at approximately 1 in 40,000–60,000 live births [6]. CDD is an X-linked disorder and exhibits a prevalence four times greater in females, indicating that germline mutations in males during the fetal period are not compatible with life [7].

In the present study, we carried out an Italian multi-center observational and retrospective study involving 14 pediatric neurology tertiary care centers to assess the phenotypic and genetic spectrum of a cohort of CDD patients. This study aims to increase the understanding of clinical and molecular aspects underlying CDD by focusing on various aspects including phenotypical presentations, seizure types, EEG patterns, treatments, neuroimaging findings, severity of psychomotor delay, and variant-phenotype correlations. We based on the assumption that gaining a deeper understanding of the overall spectrum and natural history of the CDD disease (and the associated pathogenetic variants in *CDKL5*) would be pivotal to personalized genetic counseling and clinical management of the affected children, particularly with the increasing possibilities in molecular and genetic diagnoses and, potentially, targeted treatments.

## Materials and methods

A multi-center observational study was conducted, wherein data were collected from patients with epileptic encephalopathy who were found to carry pathogenic variants in *CDKL5*. Patients were recruited at one of 14 Italian different pediatric neurology centers in Italy, with a minimum follow-up period of 3 years (except for Patient 21 and Patient 29, who were younger than 3 years). Data were collected from May 1995, until May 2023 at each center using a standardized database format, that was asked to fill in with clinical, EEG, and genetic information from both hospitalized and outpatient records. Age of onset, gender, and seizure types (at the onset and at the last follow-up evaluation) were reviewed by pediatricians, pediatric neurologists, and adult neurologists at different institutions involved in this study. Seizure types were determined and classified according to the International League Against Epilepsy (ILAE) guidelines and definitions [8]. In addition to the standardized ILAE classification, a more complete and descriptive characterization of seizures was included when available, to better identify particular sequences of epileptic seizures or peculiar semeiology, such as hyper-motor-tonic-spasms sequence (HTSS) or motor seizure patterns involving multiple phases (HTSS-like) [9, 10]. Current seizure frequency was categorized as seizure-free, monthly, weekly, and daily seizures. EEG records at onset and the last evalutation were included, with a special mention to presence or absence of hypsarrhytmia.

Other clinical data on comorbidities were collected, with particular regard to neurodevelopment and visual impairment. Physicians were asked to mention any other comorbidities, such as movement disorders, gastrointestinal problems, and sleep disturbances.

Whole-exome sequencing (WES) was performed in patients with DEEs, early-onset epilepsy, behavior disorders, and developmental delay and/or regression. The candidate variants identified in the patients from this cohort were absent from GnomAD databases and not found in control individuals within the internal genomic datasets from the participating centers involved in this study. We based on the ACMG criteria to prioritize only pathogenic or likely pathogenic. Validation and segregation studies of the candidate variants emerged by WES were performed using traditional Sanger sequencing. *CDKL5* variants were grouped according to predicted structural and functional consequences on the *CDKL5* protein as previously described [11].

Descriptive statistics were used to summarize the characteristics of individuals in the study. Statistical analysis was performed to assess whether any clinical or EEG characteristics could have prognostic significance on patients' outcomes, evaluated as seizure frequency at follow-up. Specifically, quantitative variables were analyzed using Student's t test and Mann–Whitney test based on data distribution. Conversely, the statistical methods employed for analyzing frequency tables of dichotomous data involve selecting between the chi-square test and the Fisher's exact test depending on the observed frequencies' magnitude. Statistical significance was set at *p* < 0.05. Statistical analysis was performed using SPSS 25.0 software for Windows.

## Results

We collected data of 34 individuals (30 females, 4 males) with CDKL5-related DEE, aging from 1 to 28 years. The median age at last ascertainment was 11.2 years. All patients had epilepsy and development impairment and found to carry pathogenic or likely pathogenic *CDKL5* variants. The variants were named under the canonical transcript NM_001323289.2 and confirmed de novo with traditional Sanger sequencing in all families. A summary table of clinical information, EEG findings, and genetic variants of our cohort of patients is provided in Table 1.

**Table 1:** Gene variant and main clinical features of our CDD cohort of patients

Patient	Age (year)	Gene variant [NM_001323289.2]	Age at onset (m)	Seizure type at onset	Seizure type at follow-up	Seizure frequency at follow-up
1	27.9	c.513C > G (p.Tyr171Ter)	3	FS, S, HTSS or HTSS-like	FS, S	Daily
2	20.7	c.964dupA, (p.Thr322AsnfsTer4)	1.5	TS, FS	/	Seizure-free
3	22.1	delXp22.13	2	TS, FS, S, HTSS or HTSS-like	GTCS	Daily
4	17.6	c.1090G > T (p.Glu364Ter)	3	TS, FS	FS	Weekly
5	17.9	c.176G > C (p.Arg59Pro)	10	GTCS	GTCS	Weekly
6	15.6	delXp22.13	2	FS	TS	Weekly
7	15.4	c.119C > T (p.Ala40Val)	18	FS	GTCS	Daily
8	11.6	c.2797 + 1213C > T	1	FS	FS, TS	Weekly
9	13.5	delXp22.13	2	FS	FS, TS	Daily
10	19.4	c.530A > C (p.Tyr177Ser)	2	FS	FS	Weekly
11	16.5	c.404-2A > G	0.12	FS	TS, S	Daily
12	16.7	c.104C > T (p.Thr35Ile)	1	TS, MS	TS, MS	Daily
13	14.7	c.400C > T (p.Arg134Ter)	2	MS, FS	TS, FS	Daily
14	8.8	delXp22.13	4	TS, MS, S, HTSS or HTSS-like	TS, FS	Daily
15	8.9	c.521C > A (p.Thr174Asn)	3	TS, MS, S, HTSS or HTSS-like	TS, S	Daily
16	10.1	c.1671dupA (p.Arg558ThrfsTer9)	3	TS, S	TS, GTCS	Daily
17	5.3	c.175C > T (p.Arg59Ter)	6	S	FS, S	Monthly
18	5.6	Frameshift mutation	1.5	FS, S	S	Weekly
19	1.4	Frameshift mutation	0.5	FS, S	FS, S	Daily
20	12.8	c.173del (p. Leu58TyrfsTer18)	3	S	FS, S	Weekly
21	12.1	Intragenic deletion	2	FS, S	FS, S	Seizure-free
22	4.9	Intragenic deletion	1	FS, S	FS, S	Weekly
23	4.9	Frameshift mutation	3	FS, S	FS, S	Daily
24	5.6	c.607G > A (p.Glu203Lys)	7	S	FS	Daily
25	1.2	c.400 C > T (p.Arg134Ter)	2	S	GTCS	Daily
26	4.9	delXp22.13	3	TS	GTCS, S	Daily
27	3.3	c.872G > A (p.Cys291Tyr)	7	TS	FS	Daily
28	15.6	c.863C > T (p.Thr288Ile)	9	FS	S, HTSS or HTSS-like	Daily
29	17.5	c.1006C > T (p.Gln336Ter)	1.5	TS	S	Daily
30	4.2	delXp22.13	6	GTCS	AS	Daily
31	15.2	c.587C > T (p.Ser196Leu)	1	FS	FS	Monthly
32	3.7	c.551 T > A (p.Leu184His)	1	S	GTCS	Weekly
33	3.2	c.638G > A (p.Gly213Glu)	1	FS	FS	Daily
34	3.7	Exon 1–10 deletion	3	TS, HTSS or HTSS-like	GTCS	Daily

FS: Focal seizure; S: spasms; HTSS: hypermotor-tonic-spasms sequence seizures; TS: tonic seizure; GTCS: generalized tonic–clonic seizure; MS: myoclonic seizure; AS: atonic seizure

### Epilepsy and seizure semiology

The median age of epilepsy onset was 3.4 months (range from 4 days to 18 months). About half of patients had epilepsy onset between 0 and 3 months (19/34), while the other half (15/34) had initial seizures between 3 and 18 months of life.

At the onset, most patients showed focal seizures (55.8%), followed by generalized epileptic spasms (44.1%) and tonic seizures (32.3%). Myoclonic seizures (11.7%) and generalized tonic–clonic seizures (5.8%) were less common. Focal seizures were characterized by both motor and non-motor components, and often included sudden awakening during sleep, fear, ictal eyes blinking, facial flushing, followed by asymmetrical tonic contraction or clonic seizures. Overall, 47% (16/34) of patients at the onset had more than one type of seizures among focal motor and non-motor seizures, tonic seizures, and epileptic spasms. Among patients with early-onset epilepsy (< 3 months), 73.6% (14/19) had focal motor seizures, followed by spasms in 8 patients (42.1%), and tonic seizures in 6 patients (31.5%). Whereas in cases with epilepsy onset after 3 months of life, tonic seizures were more frequent (53.3%, 8/15), as well as spasms (53.3%, 8/15). On note, generalized tonic clonic seizures were not reported in the early-onset group but in 2 of 15 patients within the group with 3–18-month seizure onset. Generalized tonic–clonic seizures, when not present at the onset, appeared later during lifetime (5.8% vs 23.5%). In our cohort of patients, HTSS or HTSS-like, even if occurring in different orders (*i.e.,* first tonic seizures, then hypermotor phase and spasms, or hypermotor phase at last) was reported in 6/34 patients (17.6%) at any lifetime point. In 5 patients, HTSS or HTSS-like appeared at the onset, whereas in one patient, they occurred in the next phases of the disease. At the last long-term evaluation, focal seizures were confirmed to be the most frequent (50%), followed by spasms (38.2%), tonic seizures (26.4%), generalized tonic–clonic seizures (23.5%), and myoclonic seizures (2.9%).

All patients initially experienced a phase of frequent seizures, occurring with weekly or daily frequency. Their response to pharmacological treatments during this period was variable and often temporary. Following this phase, a variable "honeymoon" period was observed in a large proportion of patients.

Subsequently, at the follow-up assessment, patients were categorized into four groups based on their current seizure rates, which likely indicated long-term trends in the course of epilepsy. These groups were classified as seizure-free (group 1), experiencing seizures monthly (group 2), having seizures weekly (group 3), or encountering daily seizures (group 4). At long-term follow-up, 21 patients showed daily seizures (21/34, 61.7%), 9 patients had weekly seizures (9/34, 26.4%), and 2 patients had monthly seizures (2/34, 5.8%). Only 2 patients were seizure-free at ascertainment (2/34; 5.8%). However, it is worth noting that among these two patients, one was a one-year-old girl with a relatively short follow-up period. Therefore, it may be premature to confirm that her absence of seizures was not merely a temporary honeymoon period. Overall, mean seizure rate in the group 2 was 3.0 seizures/month (ranging from 2 to 4), in the group 3 was 2, 6 seizures/week (ranging from 1 to 5), whereas in the group 4, the average frequency was 5, 4 seizures/day (ranging from 1 to 20).

None of the previously mentioned features (age of onset, type of seizure at the onset, seizure rate at follow-up) were associated with gender. The sample distribution with respect to seizure frequency did not reveal a statistically significant difference between the groups (*p*: 0.2242). The comparison between the seizure type at onset and subsequent seizure frequency did not yield statistically significant findings. Specifically, neither the presence of HTSS or HTSS-like at onset nor the occurrence of epileptic spasms showed a significant association with an increased frequency of seizures (*p* value 0.1317 and 0.8672 respectively). However, it is noteworthy that among the 6 patients exhibiting HTSS sequences at onset or later in life, all experienced multiple daily seizures. Thus, while this feature may not serve as a prognostic indicator, it could be indicative of a more severe phenotypic presentation. Subsequent extensive studies are warranted to validate this hypothesis. In addition, although this finding did not achieve statistical significance (*p* value 0.2233), among patients who subsequently developed generalized tonic–clonic seizures, all but one exhibited daily seizure occurrences.

### EEG features

EEG records showed interictal focal epileptiform discharges (spikes, polyspikes, spike wave, and polyspike wave complexes) with prevalence on the fronto-temporal or temporal–occipital regions in approximately half of the patients, with multifocal and diffuse discharges present in the remaining cases. Only 3 patients did not show EEG paroxysmal activity at the onset. EEG background activity was abnormal (slow and/or asymmetrical activity) in 20/34 cases at the onset and in 23/34 cases at the last follow-up. Hypsarrhytmia was found in 9/34 patients at the onset and in 5/34 patients at the last long-term follow-up, with all of them presenting epileptic spasms. Conversely, among the group of CDD patients who showed spasms at any lifetime point, either alone or in combination with other seizure types (16/34), hypsarrhytmia was detected in the 56.3% of them (9/16). Across the two groups of patients presenting spasms at onset, whether accompanied by hypsarrhythmia or not, there was no significant difference in daily seizure frequency (*p*: 0.4251).

### Genetic variants

Thirtyfour individuals carrying de novo variants in *CDKL5* were reported. Among these, 21/34 (61.7%) patients presented a more severe epileptic phenotype characterized by refractory epilepsy with distinct seizure types, such as tonic, myoclonic seizures, spasms, focal motor seizures, and/or hyperkinetic-tonic-spasms sequence seizures, along withdaily seizures at the follow-up. Interestingly, 8/21 (38%) of the affected patients, carried *CDLK5* deletions and/or truncations before the 172nd amino acid, with putative absence of the functional CDKL5 protein. Among these, two patients (25%, 2/8) carried the variant c.400 C > T, p.Arg134Ter. In addition, 6/21 individuals (28.5%) presented missense variants within the critical kinase domain, while 2 patients (9.5%) showed truncating variants between aminoacid 172 and aminoacid 781, and one patient (4.7%) carried a splice site variant. Individuals with a milder epileptic phenotype (4/34, 11.7%) exhibited focal motor seizures and/or spasms with a monthly frequency during the follow-up. One patient (1/4, 25%) achieved seizure control. In this group, two patients (2/4, 50%) presented with a putative truncated protein, with a termination before the 172 residue (c.175C > T p.Arg59Ter) and a gene deletion, whereas the other two individuals carried a truncating variant between amino acid 172 and amino acid 781 (c.964dupA, p.Thr322Ter) and a missense variant within the kinase domain (c.587C > T p.Ser196Leu), respectively.

### Psychomotor impairment and other clinical features

All patients showed severe psychomotor developmental delay, except for one who presented regression after a period of normal gain of psycomotor milestones (Patient 28). Cortical visual impairment was reported in all cases. The only three patients, who attained independent sitting by the age of 3 and began to develop some language abilities (Patients 3, 14, 29), were also the ones reported to have a less severe visual impairment, with intermittent eye fixation and follow, and absence of nystagmus and/or dysconjugate gaze. However, despite the milder developmental and visual imparment, they all experienced a challenging course of epilepsy with daily seizures at long-term follow-up.

Axial or generalized muscle hypotonia at the disease onset was the first sign reported by physicians in almost all the patients. In 7/34 cases (20.5%), hand stereotypes and/or dyskinesias and/or autistic features were also noticed.

Sleep disorders were also common in our cohort, as well as gastrointestinal problems, including dysphagia, gastroesophageal reflux and constipation.

### Therapeutical management

Polypharmacotherapy was required in all cases to control seizures. In total, 30 different antiseizure medications (ASMs) were prescribed. At the ascertainment, the average number of ASMs used was 2.27 (range from 0 to a maximum of 4 ASMs). In the long term, the most used ASMs were valproic acid (VPA), clobazam (CLB), cannabidiol (CBD), vigabatrin (GVG), lamotrigine (LTG), levetiracetam (LEV), and lacosamide (LCM). Among patients with the minor seizure rate (groups 1, 2, and 3), VPA alone or in combination with LEV or LCM or CLB was used. CLB associated to either LCM or LEV was used as well.

No drugs were effective at achieving a sustained seizure-free status either in monotherapy or dual-therapy. About half of the patients (16/34) received steroids (e.g., ACTH, oral prednisone, or methylprednisolone in pulse). No difference of daily seizures incidence was noted between the group treated with steroids and the group that did not received steroids (p: 0.83). Over time, several ASMs were introduced, and while some initially had a beneficial result (reduction of more than 50% of seizures) or a partial effect (reduction of less than 50%) for a limited period, they were eventually discontinued due to their inefficacy. This included medications, such as GVG, ACTH, Phenytoin (PHT), Phenobarbital (PB), Topiramate (TPM), and Methylprednisolone in pulse.

The higher percentage of withdrawal interested clonazepam (CZP) and other benzodiazepines except CLB, steroids (including methylprednisolone, hydrocortisone and ACTH), LEV, PB, TPM, and Carbamazepine (CBZ) (Table 2).

**Table 2:** ASMs used during lifetime and at last follow-up, with percentage of ASMs withdrawal

ASM	Patients treated with ASM during lifetime	Patients currently treated	% ASMs withdrawal
Clonazepam	8	0	100
Lorazepam	2	0	100
Delorazepam	1	0	100
Diazepam	2	0	100
Pyrimidone	1	0	100
Ethosuximide	2	0	100
Lacosamide	1	0	100
Buxamine	1	0	100
Methylprednisolone	2	0	100
Hydrocortisone	2	0	100
Levetiracetam	16	2	87.5
Phenobarbital	14	2	86
Topiramate	11	2	81.8
Carbamazepine	10	2	80
Nitrazepam	5	1	80
ACTH	14	3	78.6
Zonisamide	4	2	50
Felbamate	2	1	50
Rufinamide	6	3	50
Phenytoin	4	2	50
Prednisone	2	1	50
Vigabatrin	11	6	45.5
Lamotrigine	7	4	42.8
Clobazam	13	8	38.5
Perampanel	3	2	33.3
Valproic acid	19	13	31.6
Cannabidiol	7	6	14.3
Ganaxolone	0	1	0
Brivaracetam	1	1	0

ASM: antiseizure medication

In 9/34 cases, CBD was used in add on therapy. Among these patients, CBD failed to control seizures in two cases, whereas in the other patients, it caused a partial reduction of seizure rate. Neverthless, none of them were free of seizures at long-term follow-up.

Only one patient was treated with Ganaxolone, associated to TPM and LMT, and presented weekly seizures at long-term follow-up.

Across non-pharmacological treatments, KD was followed by 5/34 patients (14.7%), and it resulted effective in one patient with only monthly seizures, partially effective in 2 patients with weekly seizures and no effective in other two cases.

## Discussion

In recent years, *CDKL5* has been found to be critically involved in cell proliferation, neuronal migration, axonal outgrowth, dendritic morphogenesis, and synaptogenesis [1].

To date, at least 368 different pathogenic variants in *CDKL5* have been identified and associated with an evolving CDD-related phenotypic spectrum. The main clinical features associated with this spectrum include neurodevelopmental impairment across different domains and early infantile epilepsy with frequent drug-resistant seizures. Over the last few years, genotype–phenotype correlations in CDD have been investigated due to the higher number of individuals and disticting CDKL5 mutations identified and reported in the literature. Based on the present and the previous observations, missense variants within the critical kinase domain and truncating variants disrupting the C-terminal region (after the 781st amino acid) are usually associated with a more severe phenotype, whereas missense mutations in the ATP-binding site and/or truncating variants located earlier in the protein (between amino acid 172 and amino acid 781) have been identified in previously association to milder clinical presentations [6, 12, 13]. Interestingly, the Arg178 codon has been previously identified as a mutational hotspot for missense variants, leading to a severe phenotype with refractory epilepsy and severe neurodevelopmental impairment across all domains. Notably, a relevant phenotype variability has been highlighted, even for variants affecting close regions of the protein, suggesting that the partition in A–D groups is not optimal and rather, the impact of the single variants should be considered. Specifically, the missense variant p.Trp176Arg has been associated with a milder phenotype than the nearby variants affecting the Arg178 codon [12]. Consistently, 38% of our patients with severe refractory epilepsy carried variants previously related to a more severe phenotype. However, the same variants, putatively leading to a non-functional protein, have been found in 50% of our cohort individuals, with the mildest phenotype. Therefore, we have not observed a clear genotype–phenotype correlation in our cohort, even though it represented a small sample. Further possible contributions to the phenotypes of CDKL5 mutation carriers are the effects of modifying genes and stochastic and environmental processes during development. The broad range of epileptic phenotypes associated with CDKL5 mutations identified here is reminiscent of the variable neurological phenotypes reported in association with mutations in CDKL5 reported in the literature [12–14]. However, the kinase domain appears to be crucial in protein function, and variants variably affecting the *CDKL5* kinase activity are related to different phenotype severities. Additionally, catalytic domain missense variants and truncating mutations in the C-terminus led to differences in CDKL5 protein localization (constitutively in the cytoplasm and in the nucleus, respectively), with distinct implications on protein function and possibly severe effects on the resulting clinical phenotypes. Furthermore, variants leading to a substitution of physico-chemically divergent aminoacids could disrupt the protein function, also affecting its ability to bind substrates. Therefore, functional studies including quantitative polymerase chain reaction (q-PCR) and western blot (WB) from patient-derived tissues should be performed to precisely assess the impact of *CDKL5* variants on protein integrity and stability as a result of different mutations. The presence of mosaicism, (and its influence on phenotypical outcomes), the impact of splicing, and the random X inactivation pattern expressed in neurons fromdistinct brain regions should be investigated as well, to better investigate disease mechanism(s) and elucidate possiblegenotype-phenotype correlations in CDD resulting from individual mutations.

Importantly, early-onset drug-resistant epilepsy is the hallmark of patients with *CDKL5* mutation. In our cohort of patients, we studied the characteristics of epilepsy, with particular mention to seizure semeiology (at the onset and in the later stages of the disease) and to seizure frequency at long-term follow-up. Indeed, all patients except one were followed-up by physicians for at least 3 years from the diagnosis, allowing us to dissect the natural history of their epileptic phenotypes, without accounting for the confounding effect of the honeymoon period.

As CDD is globally considered to be drug-resistant epilepsy, we stratified patients into 4 groups according to their long-term seizure frequency: patients with good seizure control, including group 1 with no seizures (only 2 patients) and group 2 with monthly seizures (2/34); patients with intermediate prognosis including group 3 with weekly seizures (9/34) and patients with worst prognosis (group 4) that means daily seizures, from 1 to 20 seizures/day (21/34). The persistence of daily seizures (group 4) at follow-up was considered an unfavorable outcome parameter, for which we searched for possible prognostic factors at onset or clinically relevant associations.

Notably, approximately half of the patients presented seizure onset within the first 2 or 3 months of life, and this is consistent with the proportion of very early-onset CDD cases reported in different cohorts that were previously published [10, 14, 15]. However, the age at seizure onset in our cohort did not statistically relate with a higher incidence of daily seizures at follow-up appointments. Also, a late-onset of the seizures correlates with less severe epileptic presentation in terms of frequency and duration, and the patient with the latest disease onset in our cohort (Individual 7, Table 1) still experienced daily seizures poorly controlled with ASMs. Within the early-onset group (0–3 months), focal motor and non-motor seizures were the most frequent at onset, whereas in the 3–18-month-onset group, tonic seizures and spasms were more prevalent. In general, the majority of patients presented focal motor and non-motor seizures, followed by epileptic spasms, tonic seizure, and myoclonic seizures. Generalized tonic–clonic seizures were less frequent; however, they are more frequent in the later stages of the disease. The subsequent emergence of generalized epileptic seizures in patients initially devoid of such manifestations underscores a dynamic evolution within the epileptic condition. This phenomenon is shaped by a myriad of determinants with the presence of mutations in the CDKL5 gene adding an additional layer of complexity. CDKL5, involved in regulating neuronal activity and brain circuit formation, plays a significant role in epilepsy susceptibility and clinical presentation. Therefore, analyzing the evolution of seizure semiologies in patients with CDKL5 mutations requires consideration of how these specific mutations influence disease pathogenesis and progression. Disease progression remains a pivotal factor, reflecting underlying pathological changes that may lead to an expansion of epileptogenic zones or heightened neuronal hyperexcitability. Alterations in the epileptogenic substrate contribute to the shifting landscape of seizure semiologies, possibly influenced by the specific effects of CDKL5 mutations on brain development and function. Furthermore, the pharmacological efficacy of antiepileptic agents influences the clinical trajectory, as differential responses to treatment may necessitate adjustments in therapeutic regimens, potentially affecting seizure presentation. Beyond intrinsic factors, environmental and lifestyle influences continue to exert notable effects, with stress levels, sleep patterns, and exposure to potential seizure triggers serving as modulators of seizure frequency and semiology, which may interact with the underlying genetic predisposition conferred by CDKL5 mutations. Particularly, within our case series, all patients who subsequently developed generalized tonic–clonic seizures demonstrated a daily seizure frequency, except for one case characterized by weekly seizure occurrences. Approximately 40% of patients presented generalized spasms at the onset and also in subsequent periods. However, it is important to note that the presence of spasms did not correlate with a worse prognosis compared to other seizure semiologies.

The most characteristic feature found in CDD was the presence of a variety of epileptic seizure patterns, which may occur sequentially throughout during lifetime (e.g., onset with spasms, later stages characterized by tonic or focal seizures) or coexist in a given phase of the disease. In fact, about half patients already showed more than one seizure type between focal, tonic, and spasm seizures at the onset of the disease. In some cases, different seizure types could even occur simultaneously in the same electroclinical event, as in the case of HTSS, a sequence characterized by hypermotor seizures, tonic seizures and epileptic spasms, or by analogous sequences described as HTSS-like, in which we can find these 3 phases in different order or just the tonic seizure and spasms clustering together. According to our experience, patients presenting with HTSS or HTSS-like at onset or later in life were characterized by a higher incidence of multiple daily seizures at long-term follow-up. However, due to the overall severity of the CDD and the small sample size, this comparison did not achieve statistical significance. Nevertheless, our experience still leads us to hypothesize that such sequences could serve as potential indicators of a more severe phenotype. Indeed, the greater phenotypic variability in seizures among patients with CDD may reflect a more pronounced involvement of the Central Nervous System (CNS) and, consequently, a higher prevalence of drug-resistant epilepsy among them. Unfortunately, due to the small sample size of males with CDD in our cohort, the phenotypic variables analyzed in this study did not show any correlation with gender.

Regarding EEG features, all patients showed focal, bifocal, or multifocal paroxysmal discharges at the onset or later during lifetime. About 26% of patients showed hypsarrhythmia (classical or modified) at the onset or in subsequent periods and all of them presented spasms alone or in association with other kind of seizures. However, among patients with spasms (16/34), hypsarrhythmia was reported in approximately 60%, a slightly higher value compared to what reported by Demarest and colleagues (47%), but still lower compared to children with infantile spasms of other etiologies (82%) [16]. A recently published study on epileptic spasms in children with CDD found that a poorer response to treatment among CDD patients with spasms was associated with delayed treatment initiation [17]. However, in our case series, no EEG abnormalities, including hypsarrhythmia, were found to correlate with seizure frequency at follow-up.

As we expected, all the cases included in our study showed moderate to severe psychomotor development delay, except one case of developmental regression after the initial achievement of milestones, underlining the difference with Rett syndrome [18, 19].

Similarly, all CDD patients had a variable degree of CVI. Due to the observational nature of the study, it was not possible to standardize the collection of neurodevelopment information and the evaluation of CVI degree for a statistical evaluation. However, from the descriptive data collected, we noted that the only three children with a milder developmental delay (moderate) were the only ones to present a lower degree of visual involvement, with the presence of eye fixation (even if intermittent), eye follow, and absence of nystagmus or dysconjugate gaze. The prognostic value of CVI had already been postulated in the 2019 by Demarest and colleagues, who demonstrated a correlation between the degree of CVI and the severity of developmental impairment, but they did not study the relationship with the epilepsy course [10]. A previous work found out that the ability to walk and use of language were associated with lower rates of current seizures, regardless of the gene variant [3]. Conversely, in our case series, the 3 patients with the least visual involvement and milder psychomotor delay, showed daily current seizures, suggesting an independent trend of these two variables. Obviously, further correlation studies are needed to clear this relationship. To better compare results, it would be necessary using psychomotor development assessment scales specific for CDD that take into account the slow, albeit constant, achievement of milestones during lifetime, as well as to better assess CVI degree, standardized clinical data (i.e., eye fixation and follow-up, nystagmus) should be accompanied with instrumental information (visual evoked potentials).

Our experience confirmed the drug-resistant nature of CDD [20]. No ASM could be considered effective in achieving seizure freedom, neither as monotherapy nor in add on, as expected from literature [21]. In our cohort, up to 30 different ASMs have been attempted to control seizures. At the time of the last evaluation, the most used ASMs were VPA, CLB, CBD, GVG, LTG, LEV, and LCM. The higher percentage of withdrawal interested CZP and other benzodiazepines except CLB. This trend was noted even for medications that had limited and temporary efficacy, such as VGB, ACTH, PHT, PB, TPM, and methylprednisolone in pulse. Interestingly, among our patients, CBD seemed to have a partial effect although none of patients achieved seizure freedom after CBD administration. However, confirming its effectiveness, CBD had the lower withdrawal percentage at ascertaiment compared to the other ASMs.

## Conclusions

This Italian multi-center observational study adds one more piece to the expanding knowledge of epilepsy in CDD. The originality of this study lies in its description of a new case series aimed at assessing the natural history of patients with CDD. Particular attention was focused on correlational analysis between seizure semiology and seizure frequency at follow-up, aimed at identifying any quantitative or qualitative variables that may guide prognostic assessment of these patients. Based on our case series, it is not feasible to delineate clinical or EEG features as prognostic indicators of seizure severity. However, among our cohort, patients who exhibited the emergence of generalized tonic–clonic seizures over time were characterized by a heightened frequency of seizures during follow-up. Furthermore, it is worth noting that all 6 patients, who exhibited HTSS or HTTS-like sequences at onset or later in life, presented with a severe phenotype, characterized by multiple daily seizures. A larger sample size in future studies could potentially lead to achieving statistical significance and confirming this observation. Further studies are needed to better characterize the relationship between the degree of psychomotor development delay and cortical blindness and the course of drug-resistant epilepsy in these patients. Last but not the least, functional studies aimed to characterize the impact of CDKL5 mutations from patient-derived cell types (including brain organoids) will be pivotal to establish the impact of mutations and to dissect individual disease mechanism(s), to explain the broad heterogeneity in terms of neurodevelopmental and epileptic presentations of the affected patients and to assess the influence of additional genetic and non-genetic factors on phenotypical outcomes of CDD.

## Data Availability

Not applicable.
